# Research progress on rheumatoid arthritis-associated depression

**DOI:** 10.3389/fnbeh.2022.992223

**Published:** 2023-01-23

**Authors:** Nian Liu, Weitian Yan, Rong Su, Lin Zhang, Xingqiang Wang, Zhaofu Li, Dongdong Qin, Jiangyun Peng

**Affiliations:** ^1^First Clinical Medical School, Yunnan University of Chinese Medicine, Kunming, China; ^2^Rheumatism Center, Yunnan Provincial Hospital of Traditional Chinese Medicine, Kunming, China; ^3^Basic Medical School, Yunnan University of Chinese Medicine, Kunming, China

**Keywords:** rheumatoid arthritis, depression, etiology, pathology, biological therapies, research progress

## Abstract

Depression is an independent mood disorder and one of the most common comorbidities of rheumatoid arthritis (RA). Growing evidence suggests that there is two-way regulation between RA and depression, resulting in a vicious cycle of RA, depression, poor outcomes, and disease burden. The rising prevalence of RA-associated depression warrants a re-examination of the relationships between them. Here we provide an overview of the etiology and pathological mechanisms of RA-associated depression, and recent advances in treatment with biologics, which will facilitate the development of new and effective prevention and treatment strategies.

## 1. Introduction

Rheumatoid arthritis (RA) is a systemic autoimmune disorder characterized by synovitis, joint erosion, and cartilage damage. In the global burden of disease study in 2010, the disability imposed by RA ranked 42 among the 291 diseases included (Cross et al., 2014). In addition to the disability caused by joint pain, swelling, and deformation, the extra-articular symptoms of RA also require attention. Depression is a mood disorder affecting 322 million people worldwide, and one of the most common comorbidities in RA (World Health Organization, 2017; Baerwald et al., 2019). Depression can be triggered by multiple stimuli such as repeated physical pain, fatigue, gradual loss of function, lack of social role, and financial burden. In a cross-sectional study conducted in 17 countries, depression was the most frequent complication in RA, with prevalence ranging between 14% and 48% (Nerurkar et al., 2019). In China, the proportion was as high as 48%, and the respective prevalence of mild, moderate, and severe depression was 30%, 18%, and 18% (Fu et al., 2017). Although the prevalence of depression in RA patient groups varies in different countries and regions due to measurement methods as well as the non-uniform threshold of diagnostic criteria for major depressive disorder (MDD; Sturgeon et al., 2016), it also reminds us that depression is a link that cannot be ignored in RA treatment. This article reviews the research progress on RA-associated depression from: (1) etiology; (2) pathology; and (3) biological therapies, hoping to provide a reference for future basic and clinical research on RA-associated depression.

## 2. What causes depression in RA patients?

In 1977, Professor Engel of the Medical School of the University of Rochester put forward a new medical model, the bio-psycho-social medical model. This new model emphasized the combination of biology, psychology, and sociology to search for the causes, diagnosis, and treatment methods of diseases, instead of investigating diseases from a single biomedical perspective. Given the debate as to why RA is often associated with depression, more complex and comprehensive factors covering biological, psychological, and sociological needed to be considered rather than a simple causal model of psychological impairment due to chronic pain and long-term disability associated with RA.

**Figure 1:**
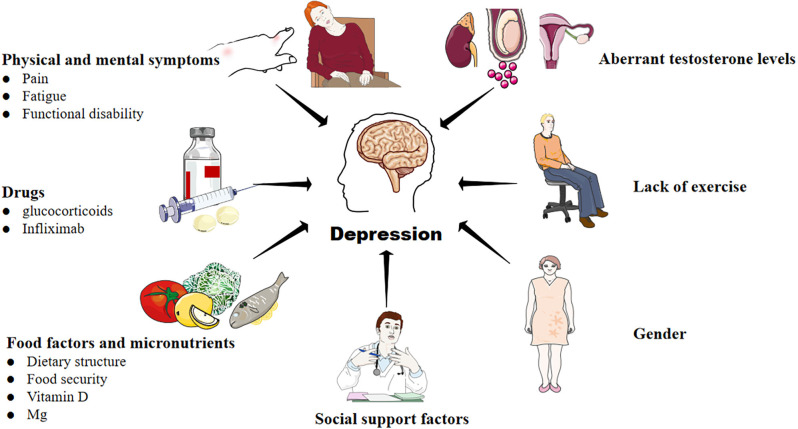
Factors that contribute to the development of depression in RA. A variety of complex and comprehensive factors, including physical and mental symptoms, drugs, food and micronutrients, lack of exercise, aberrant testosterone levels, and social support may contribute to depression in RA.

There is a bidirectional association between RA and depression. On the one hand, under the influence of pain, fatigue, drugs, diet, micronutrient, gender, lack of exercise, aberrant testosterone levels, and social support (including social tool support, emotional support, and financial assistance), RA patients often face poor health-related quality of life, reduced chance of joint symptom relief, and a higher risk of death (Marrie et al., 2018; Shadick et al., 2019; Vallerand et al., 2019; Lwin et al., 2020; Figure 1). They have to overcome more severe obstacles in maintaining biological function, mental health, as well as social participation. As a result, the risk of depression in RA patients is significantly higher than in non-RA groups (Lin et al., 2015; Lu et al., 2016; Marrie et al., 2018). On the other hand, RA patients associated with depression will bear “overload hospitalization costs” due to more physician visits, increased emergency care utilization, and the use of more drug types to treat depression (Hitchon et al., 2021). Thus, some patients have to reduce the cost of RA treatment, which aggravates RA. In a word, RA is a risk factor for depression, and depression can exacerbate the severity of RA. The two diseases fed on each other, pushing the patients into a vicious cycle of “RA-depression-adverse outcomes-social and economic burden”.

### 2.1. Physical and mental symptoms

Pain is the most typical symptom of RA. Even if the inflammation has been controlled, patients often experience chronic pain. A German cross-sectional study based on data from nationwide statutory health insurance fund (BARMER GEK) reported that depressive symptoms were far more likely to develop in RA patients with severe pain (75.3%) than in those with moderate pain (53.1%) or mild/no pain (21.0%; Jobski et al., 2017).

Fatigue is a common mental symptom in RA patients. Approximately one-sixth of RA patients experience severe fatigue, which is related to pain, personality characteristics, gender, sleep, social support, and comorbidities (Nikolaus et al., 2013). In addition, it is affected by drugs such as methotrexate (Pope, 2020). A study investigating risk factors for depression and deterioration of depressive symptoms in 2018 indicated that depression and depressive symptom deterioration in RA positively correlated with the degree of fatigue [odds ratio (OR) 1.26] (Cheon et al., 2018). These results suggest that doctors need to pay more attention to the possibility of depression for RA patients who are prone to fatigue symptoms.

### 2.2. Drugs

The finding that recurrent depressive disorder without antidepressant treatment is a significant predictor of the progression of joint destruction in RA suggests both RA and depression have to be taken into account in the treatment for RA-associated depression (Abramkin et al., 2020). Similarly, it is necessary to be aware of the possibility of depression caused by the drugs for RA treatment. Among the drugs currently used to treat RA, glucocorticoids (GCs) and infliximab need attention. GCs have a series of biological effects, including anti-inflammation, immunosuppression, regulation of metabolism, and cognitive signal transduction (Scherholz et al., 2019). Exogenous GCs supplementation is a conventional treatment for RA. Long-term exposure to exogenous GCs can also cause some severe adverse effects however, such as infection, osteoporosis, cushing syndrome, and some emotional disorder symptoms including depression (Pamukcu et al., 2021). A German study compared the physical condition of RA patients whose daily dose of prednisone exceeded 0.5 mg in the past 6 months with others that had not received any GCs therapy in the past 12 months. The results showed that a daily dose of prednisone exceeding 7.5 mg was a threshold for a significant increase in the frequency of depression (Huscher et al., 2009). Through the study of the macaque rhesus model of depression, the mechanism of depression induced by chronic GCs exposure was found to be related to the decrease of hypothalamic-pituitary-adrenal (HPA) axis cortisol level in blood, the increase of hair cortisol concentration, and the decrease of dopamine level in cerebrospinal fluid (Qin et al., 2019).

Another drug with a high possibility of causing depression is infliximab, the first tumor necrosis factor α (TNF-α) antagonist used to treat chronic inflammatory diseases, and characterized by rapid therapeutic effect and high bioavailability. The up-regulation of TNF levels in depressed patients has been demonstrated, therefore, infliximab is used to treat depression also (Rani et al., 2022). In 2014, a clinical study involving 34 RA-associated depression patients showed that infliximab could reduce RA disease activity and improve symptoms of depression (Miwa et al., 2014). However, randomized controlled trials conducted in Canada and the United States showed that infliximab did not significantly reduce depression in adults with bipolar depression compared with a placebo (McIntyre et al., 2019). Infliximab is ineffective in reducing depressive symptoms when used for treatment-resistant depression, which is significantly related to TNF levels. Furthermore, according to the research of the Thillard team, which enrolled 118,528 RA patients, the hazard ratio of developing depression associated with infliximab exposure is 3.49 (Thillard et al., 2020). Compared with infliximab, RA patients who received etanercept had a lower risk of depression, and it is suggested that etanercept may be the more appropriate biologic drug for RA-associated depression (Ng et al., 2020). Although there is still a lack of high-quality research evidence on the risk of depression caused by RA treatment drugs, it is undeniable that the existing evidence may still help clinicians adjust the choice of drugs and improve the benefit-hazard ratio.

### 2.3. Vitamin D and magnesium deficiency

Vitamins and minerals in a healthy body are maintained at a relatively constant concentration, involved energy metabolism, DNA synthesis, oxidative stress, and neuronal function to support the normal function of bone, muscle, and brain. Once the stability of this concentration is broken, it means the possibility of disease (Tardy et al., 2020). Recent studies have shown that vitamins, magnesium (Mg), zinc, selenium, copper, and other trace elements with antioxidant effects are involved in RA inflammation, whereas research on RA-associated depression has mainly focused on vitamin D and magnesium.

Vitamin D is a fat-soluble vitamin that can bind to vitamin D receptors in different tissues and cells, and plays an essential role in calcium homeostasis and bone metabolism (Sizar et al., 2022). Evidence suggests that vitamin D can also affect mental health (Föcker et al., 2017). In a 2017 study of 161 RA patients, serum vitamin D levels in those with depression were significantly lower than those without depression, and vitamin D levels were negatively correlated with Hamilton Depression Scale scores and Hamilton Anxiety Scale scores (Pu et al., 2017). The results suggested that vitamin D deficiency may be a risk factor for depression in RA patients.

Mg is an antioxidant micronutrient to improve the function of antioxidant enzymes and reduces inflammatory conditions. 50%–60% of Mg is stored in bone tissue to maintain bone health, and relieve chronic musculoskeletal pain in RA (Arablou et al., 2019; Capozzi et al., 2020; Elma et al., 2020). In terms of brain biochemistry, patients with depression show abnormal glutamate and gamma-aminobutyric acid (GABA) neurotransmission. Mg can increase the expression of GluN2B, a subunit of the glutamatergic n-methyl-D-Aspartate receptor (NMDAR), and inhibit the phosphorylation of eukaryotic elongation factor 2 (eEF2) in cells, antagonize the NMDAR to affect the transmission of glutamate and other neurotransmitters, resulting in antidepressant effects (Górska et al., 2019). Although Mg plays a vital role in the regulation of both inflammation and brain biochemistry, its efficacy in the treatment of RA-associated depression remains controversial. Cross-sectional studies indicated that the dosage of Mg on diet was inversely associated with the risk of RA and depression (Sun et al., 2018; Hu et al., 2020). RA prevalence was kept to a minimum when Mg intake was between 181 and 446 mg/day, and the risk of depression was reduced at 320 mg/day (Li et al., 2017; Hu et al., 2020). However, a prospective study in the SUN Mediterranean cohort with an expanded sample size of 15,836, and an extended follow-up (median = 10.2 years) study confirmed that no significant association between Mg intake and low risk of depression (OR = 0.85, 95% CI: 0.60–1.22; Martínez-González and Sánchez-Villegas, 2016). In addition, low Mg intake has been identified as a protective factor in reducing the risk of depression in older adults (Tarleton and Littenberg, 2015). These results may be influenced by the uncertainty and complexity of causality in cross-sectional studies. More prospective studies are needed to evaluate the effect of magnesium on RA-associated depression in the future.

### 2.4. Exercise

Sedentary behavior is prevalent in RA patients due to impaired physical function and persistent fatigue (Fenton et al., 2018). Overwhelming data indicate that exercise treatment has a therapeutic effect on various chronic diseases involved in many systems, including the neuropsychiatric system, endocrine system, cardiovascular system, and musculoskeletal system, among others. Reductions in daily physical activity can lead to impaired functionality and premature damage to health (Booth et al., 2012; Pedersen and Saltin, 2015). For RA patients, any exercise will get more clinical benefits than no exercise, whether it is hand exercise, rejoice exercise, Taichi, strength training, aquatic exercise, resistance exercise, or cryotherapy (Hu et al., 2021).

Exercise also has a therapeutic effect on depression. Studies have found that exercise and antidepressant drugs can increase the secretion of brain-derived neurotrophic factor (BDNF), serotonin, and norepinephrine, enhance the activity of the HPA axis, reduce systemic inflammatory signals to promote the development of new neurons, and strengthen synaptic connections between neurons to alleviate depression. In addition, exercise changed the structure of the hippocampus, anterior cingulate, orbitofrontal cortex, and enriched the blood vessels of the brain (Gujral et al., 2017). Aerobic exercise and/or strength training can significantly relieve depressive symptoms in adults with arthritis and other rheumatic diseases (Kelley et al., 2015). Tai Chi (Waite et al., 2013), Pilates (Yentür et al., 2021), yoga (Bosch et al., 2009), and medium-high intensity exercise (Kucharski et al., 2019) were also influential. What calls for special attention is that some patients may have limited exercise patterns due to arthritis or functional impairment. To maximize the benefit, the intensity, frequency, and cycle of exercise should be formulated according to individual symptoms and wishes. It poses a greater challenge to the professionalism of healthcare personnel and the improvement of social movement facilities.

### 2.5. Diet

The involvement of dietary structure in the pathogenesis of depression in general population has long been confirmed by clinical studies. In 2021, scholars introduced this concept into the field of RA, and results showed that the eating habits of RA patients were also associated the occurrence of depression. It has been demonstrated that among 20 foods, including vegetables, grains, meat, fish, and fruits, intake of fish, vegetables, and fruits were inversely related to depression scores in RA patients with frequent intake of fish (≥3 times per week). Improving eating habits, especially increasing intake of fish may contribute to alleviating depression in RA patients (Minamino et al., 2021). In addition to dietary structure, food source security is highly correlated with the development of depression in RA, and their correlation becomes much more conspicuous as soon as food safety declines. Compared to RA patients with complete food security, food-insecure patients had a significantly higher risk of depression (OR = 2.96, 95% CI: 1.48–5.90; Cai et al., 2022). Once food insecurity is improved, the statistical significance of the correlation gradually declines.

### 2.6. Gender

Patients with RA are at greater risk for severe depression than gender-matched healthy individuals (Khan et al., 2021), and the risk within the RA group is reflected in the higher risk of depression in females compared to males (Albrecht, 2014; Kim et al., 2020). In addition, there are also gender-related differences in the causes of severity of depression among RA patients. According to McQuillan et al. (2022), RA functional disability is more strongly associated with depression in males than in females. Depressive symptoms in female patients appear to be more closely related to poor sleep quality or family pressure (Hughes et al., 2021; Hamasaki et al., 2022).

### 2.7. Aberrant testosterone levels

Testosterone is a sex hormone synthesized in the gonads and the adrenal gland. In genera, testosterone levels are significantly higher in men than in women. Previous studies have shown that testosterone has an immunosuppressive effect, which can inhibit the onset of RA to an extent. A decline in testosterone level is related to RF-negative RA, and may also induce depression (Pikwer et al., 2014; Gubbels Bupp and Jorgensen, 2018; Walther et al., 2019; Maharjan et al., 2021). However, there is also evidence that excessive testosterone can have adverse effects on mental and physical health. In a two-sample Mendelian randomization study conducted in 2021 abnormally high testosterone level is associated with a risk of RA and depression (Syed et al., 2020). Thus, aberrant fluctuation of testosterone may contribute to RA and depression.

### 2.8. Social support

Social tools and social emotional support are independent factors affecting the severity of depression in RA. Based on DAS28 score, an analysis of psychosocial characteristics in RA patients with and without remission showed that emotional support had a significantly beneficial effect on the severity of depressive symptoms in RA in remission, whereas instrumental support had an extremely limited effect. In the non-remission group, the positive regulatory effect of instrumental support was relatively significant, and emotional support was also helpful for depression (Yasuoka et al., 2021). The results indicated that the treatment of RA-associated depression should not focus solely on the medical control of disease activity by doctors, but should also recognize the need for social support to cover instrumental and emotional to improve overall physical and mental wellbeing (Khan et al., 2021).

## 3. Pathology of interaction between RA and depression

Although the mechanism of interaction between RA and depression is still unclear, some previous findings have provided insight into directions for further investigation. As an immune-mediated inflammatory disease, RA has associated the abnormal expression of pro-inflammatory and anti-inflammatory mediators induced by an imbalance in immune tolerance. Similarly, depression is associated with abnormal activation of the immune system and inflammatory responses (Beurel et al., 2020). Depressed patients are likely to exhibit increases in neutrophil/lymphocyte, platelet/lymphocyte, monocyte/lymphocyte ratios (Marazziti et al., 2021), and a shift from classical monocytes toward non-classical monocytes (Hasselmann et al., 2018). Serum interleukin 6 (IL-6), TNF, and C-reactive protein (CRP) were also higher in depressed patients than in a healthy control group (Beurel et al., 2020). The levels of IL-6 and TNF in cerebrospinal fluid, and translocator protein (PET marker of central inflammation) in the anterior cingulate cortex and temporal cortex are higher in MDD patients when compared to controls, suggesting that central inflammation may be involved in MDD (Enache et al., 2019). Notably, there is significant heterogeneity in the levels of circulating inflammatory factors in patients with depression, and this heterogeneity is also reflected in responses to antidepressants (Liu J. J. et al., 2020). For example, increased CRP is seen in resistant MDD, rather than in depressed patients generally (Chamberlain et al., 2019). It seems that as well as immune disorder and inflammation, other mechanisms are also involved in the interaction between RA and depression.

### 3.1. Immune inflammatory stimulation

The stimulating effects of peripheral inflammation in RA on the central nervous system (CNS) are regarded as the main triggering mechanism of depression. The inflammatory bias is one of the most important mechanisms that connect the two diseases. A genetically-based inflammatory bias that arose during early human evolution is critical for humans to fight infection, heal wounds, and maintain vigilance to attack. This inflammatory bias is suppressed by regulatory T (Treg) cells, regulatory B (Breg) cells, and immunoregulatory M2 macrophages, as well as the anti-inflammatory cytokines interleukin-10 (IL-10) and transforming growth factor β (TGF-β) in the rural environment. In modern society, psychological challenges have been increasing along with the decline in infectious challenges that have left the former immune checks and balances lacking. These psychological challenges stimulate the overproduction of inflammasome in myeloid cells, which mediates responses to non-pathogenic or “sterile” stressors and leads to the development of a variety of disorders, including depression (Miller and Raison, 2016). In previous studies, the activation of inflammasome also plays a crucial role in immune dysregulation and joint inflammation (Jiang et al., 2022). NLRP3 inflammasome expression in the synovium is increased in collagen-induced arthritis (CIA) model, and targeted inhibition of NLRP3 activation, contributes to inhibiting the progression of RA (Zhang et al., 2016; Liu P. et al., 2020).

Peripheral inflammatory signals can reach the brain through humoral and neural pathways. There are three types of humoral pathways. First, pro-inflammatory cytokines can cross the blood-brain barrier (BBB), and contact the brain *via* periventricular organs and the choroid plexus. Second, TNF and other inflammatory mediators bind to cytokine receptors on the membranes of cerebrovascular endothelial cells directly, activate a second messenger, are transported into the CNS, which leads to the activation of microglia and the subsequent secretion of pro-inflammatory factors in the brain. Third, blood-derived immune cells and pro-inflammatory cytokines can access the brain through a damaged BBB (Süß et al., 2020). A recent study found that microglia in the area postrema (a brain region lacking a BBB) significantly increased in density and kept highly activated during persistent autoimmune arthritis, which demonstrates that chronic inflammation in RA may affect microglia in brain regions lacking a BBB and result in CNS-mediated symptoms, such as depression (Matsushita et al., 2021).

In the neural pathways, pro-inflammatory mediators stimulate active primary afferent nerves to transmit peripheral inflammation to the CNS (Fakra and Marotte, 2021). The immune system and nervous system can communicate with each other mainly depending on the activation of the HPA axis by pro-inflammatory cytokines and afferent vagal fibers. Cytokines also directly impact the cerebral cortex and nuclei in the brain stem (Ingegnoli et al., 2020). How the vagus transduces inflammatory signals to the CNS and causes depression is still not fully understood. Vagus nerve stimulation is routinely used in the clinic to treat depression. It has been shown that severing the connections between the nucleus of the solitary tract (NTS) and the higher brain regions can reduce stimulation-induced activation for NTS neurons receiving myelinated vagal input, suggesting that higher brain regions play a significant role in maintaining both regular activity in NTS and indirect mechanisms of enhancing NTS neuronal activity during vagus nerve stimulation (Cooper et al., 2021). The study indirectly explains the vagal pathway that transmits inflammatory signals to the CNS. Once the level of inflammatory cytokines in the CNS increases, activated indoleamine 2,3-dioxygenase may enhance tryptophan catabolism, inducing serotonin depletion and kynurenine production. Kynurenine is then further transformed to 3-hydroxykynurenine and quinolinic acid, which can lead to an elevated glutamate level and oxidative stress response, reduce GABAergic inhibitory control, and cause apoptosis in the hippocampal and medial prefrontal cortex (Belleau et al., 2019). Previous studies have also shown that IL-1β and TNF reduced serotonin levels by activating serotonin transporters, ultimately causing a depressive state (Zhu et al., 2006; Malynn et al., 2013). Furthermore, pro-inflammatory cytokines can affect synaptic plasticity and neurogenesis by reducing the expression of brain-derived neurotrophic factors, resulting in structural and functional alteration of the brain (Calabrese et al., 2014). Cortical neural circuits involved in emotion and stress regulation trigger depression under the above triple stimuli (Fakra and Marotte, 2021). The over-active HPA axis, which is wildly associated with depression, can also be induced by pro-inflammatory cytokines. Adzic et al. (2015) have already shown that depressive-like behavior caused by lipopolysaccharide-inducing peripheral inflammation in rats emerges from HPA axis activation and sex-specific alterations of hypothalamic molecular signaling. Interestingly, it is found that MDD can in turn trigger pro-inflammatory shifts in monocyte subsets and decrease the expression of steroid signaling-related genes (Hasselmann et al., 2018). Under stress, upregulated calcium/calmodulin-dependent protein kinase II in the hippocampus will promote the transcription and expression of cyclooxygenase-2, enhance the level of the pro-inflammatory factor prostaglandin E2, and aggravate RA joint synovial inflammation (Vallerand et al., 2019). Furthermore, depression is associated with the overactivation of the sympathetic nervous system (SNS; Bucciarelli et al., 2020). It has been demonstrated that RA patients frequently have an unbalanced autonomic nervous system, with decreased parasympathetic and increased sympathetic tone (Koopman et al., 2011). In a murine model of lymphoproliferative disease, the SNS induces apoptosis in immunosuppressive CD4(+) Foxp3(+) regulatory T cells, which suggests overactive SNS driven by depression can lead to RA *via* peripheral immune activation (Wirth et al., 2014).

### 3.2. Signal pathways

In the bi-directional feedback between RA and depression, the transduction of immune and inflammatory signals inside and outside cells is mainly completed by JAK/STAT and MAPK signal pathways. JAK/STAT is a rapid membrane nuclear signal module composed of transcription factors of the Janus kinase family and the STAT family, which regulate the pathological and physiological processes of RA by mediating interferon (Villarino et al., 2017). The JAK/STAT pathway is driven by pro-inflammatory cytokines, leading to elevated expression of the matrix metalloproteinase gene, accelerated chondrocyte apoptosis, and decreased apoptosis resistance in inflamed synovial tissue, which plays a critical role in the development of RA (Malemud, 2018). Cytokines can also activate indoleamine 2,3-dioxygenase in glial cells by stimulating STAT1, leading to a reduced source of serotonin production and subsequent depression (Yan et al., 2018). MAPK is a group of threonine/serine protein kinases that transduce extracellular stimuli to the nucleus. In RA, inflammatory factors activate the MAPK signaling pathway, causing synovial tissue proliferation and joint destruction. It can also accelerate the clearance of serotonin in synapses *via* the p38 MAPK signaling pathway, enhance glucocorticoid resistance, cause synaptic plasticity imbalance, and ultimately lead to depression (Malemud and Miller, 2008).

### 3.3. Oxidative stress

Oxidative stress is a pathological state of redox imbalance caused by increased production of reactive oxygen species (ROS) and/or decreased antioxidant capacity (Salim, 2017), which produces free radicals that act as oxidants and inflammatory mediators involved in RA pathology. Excessive ROS in RA patients can reduce the function of free radical enzyme defense systems, lead to a rapid increase in free radical levels, aggravate weakening effects on the hippocampus, amygdala, and cortex connection, and eventually accelerate the occurrence of depression (Bala et al., 2017; Salim, 2017). Alouffi et al. (2018) found that compared to patients with RA alone, levels of carbonyl (a protein oxidation marker mediated by ROS) were higher in patients with RA and depression. It is speculated that inhibiting the process of oxidative stress in RA will help to reduce the probability of RA-associated depression, or alleviate the degree of depression.

### 3.4. Central sensitization and pain

**Figure 2:**
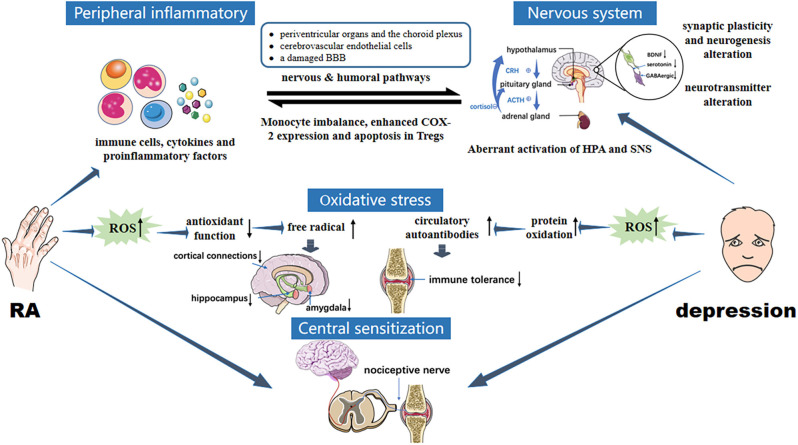
Pathology of interaction between RA and depression. RA immune tolerance imbalance induces abnormal expression of inflammatory mediators, activated peripheral inflammatory signals enter the brain through humoral and neural pathways. CNS inflammation is induced, as are overactivation of HPA, changes in brain structure and function, upregulation of glutamate levels, reduced GABA expression and brain-derived neurotrophic factors, enhanced oxidative stress, and increased ROS levels, leading to depression. In the two-way feedback between RA and depression, the transduction of immune and inflammatory signals in and out of cells mainly involves JAK/STAT and MAPK signaling cascades. In addition, central sensitization enhances pain perception and aggravates depression under the stimulus of chronic inflammation in RA. Oversensitivity caused by depression in turn exacerbates pain, creating a vicious circle between RA and depression.

Pain is not only the leading cause of the medical behavior of RA, it is also strongly associated with the occurrence of depression (Lwin et al., 2020). Recent studies have shown that the pain symptoms in RA are co-regulated by both the peripheral nervous system and the CNS (Harth and Nielson, 2019). In the pathological process of RA, adaptive and innate immune systems are activated, producing a series of inflammatory mediators. Then, neutrophils, T lymphocytes, and B lymphocytes are driven into the synovium, leading to local synovial inflammation. In the inflammatory environment, fibroblast-like synovial cells secrete nerve growth factors and upregulate the release of substance P, neuropeptide, kinin, IL-6, TNF, and other molecules, sensitizing the nociceptor terminals of inflammatory periarticular tissues and primary afferent neurons, resulting in the production of pain (Walsh and McWilliams, 2014). Central sensitization in the spinal dorsal horn of the cerebrospinal fluid expands and enhances pain perception in the sensory area. Remodeling of inflammatory joint nerve fibers may also contribute to the generation and maintenance of arthritis pain (Gonçalves Dos Santos et al., 2020). Inflammation, the central source of pain in RA, is also closely related to non-inflammatory factors. Researchers have revealed that cytokines can directly cause central sensitization through the nociceptive nervous system, and reduce the pain threshold, resulting in persistent pain (Schaible, 2014; Sebba, 2021). Although there is no direct correlation between depression and central sensitization, patients with depression are more sensitive to psychological and physical pain than patients without depression (Conejero et al., 2018; Figure 2). In RA patients, chronic inflammation impairs physiological stress resistance and effective coping behavior, leading to depression. Hypersensitivity caused by depression will also undoubtedly aggravate pain, and indirectly promote the deterioration of RA. Clinicians should be mindful that anti-depressants is considered if pain symptoms persist after early, standardized, combined DMARDs, NSAIDs, and GC treatment, if patients have achieved remission but still experience joint pain based on the DAS28 score (Zhang and Lee, 2018).

## 4. Biological therapies for RA-associated depression

The treatment of depression mainly includes drug therapies, psychological intervention, and comprehensive nursing. In a study conducted by Yasuoka et al. (2021), the depressive symptoms of RA patients in remission (DAS28 score < 2.6) could be significantly improved by emotional support, but whether this applies to non-remission patients remains uncertain. From this study, there is general uncertainty about the efficacy of non-pharmacological therapies for depression with RA. With the role of cytokines in the pathological mechanism of RA-associated depression gradually being discovered, the value of biological agents in the treatment of RA and depression has become a hot research topic (Table 1).

**Table 1:** Biotherapies for RA-associated depression.

Research type	Intervention	Number of subjects	Time	Results	Reference
Randomized control trial	bDMARDS vs. csDMARDS	90	24 w	bDMARDs were not superior to csDMARDs with regard to their effects on anxiety and depression in patients with RA.	Yayikci and Karadag (2019)
Randomized control trial	bDMARDs vs. MTX vs. LEF vs. HCQ	105	unclear	RA with bDMARDs had the high rate of depression compared with MTX, LEF, and HCQ.	Pinho de Oliveira Ribeiro et al. (2013)
Self-control trial	bDMARDS	18,241	48 w	Depressive symptoms improved by 20%–40% after 1 year of biologic therapy, but preexisting depressive symptoms at the time of receiving the first biologic may reduce the chance of treatment response.	Matcham et al. (2018)
Retrospective study	bDMARDS	152	24 w	Younger patients with lower depression scores at baseline can achieve depressive remission with bDMARDs.	Miwa et al. (2018)
Retrospective observational cohort study	Anti-TNF	4,222	48 w	RA patients who responded to anti-TNF had significantly lower risk of depression	Deb et al. (2019)
Prospective cohort study	Anti-TNF vs. MTX	1,326	48 w	Depression was a strong negative predictor of disease remission in patients with RA after 3 and 6 months of anti-TNF or MTX treatment.	Michelsen et al. (2017)
Systematic review and meta-analysis	Infliximab vs. placebo	152	12 w	There was no statistically significant effect of infliximab as an adjuvant treatment for treatment-resistant depression.	Bavaresco et al. (2020)
Retrospective cohort study	Infliximab	7,600	unclear	Infliximab treatment increased the risk of adverse events of mental illness, and may increase suicidal tendencies in RA patients.	Thillard et al. (2020)
Case control study	Tocilizumab vs. non-biological systemic treatments	82	4 w	High IL-10 in RA is associated with an increased risk of depression, tocilizumab can reduce depressive symptoms.	Figueiredo-Braga et al. (2018)
Multi-center, single-arm study	Tocilizumab	91	24 w	Tocilizumab treatment may be significantly associated with improvement RA-associated depression.	Tiosano et al. (2020)

Note: This table summarizes research of bDMARDs for the treatment of RA-associated depression. HCQ, hydroxychloroquin; LEF, leflunomide; MTX, methotrexate; w, weeks.

### 4.1. bDMARDs

Variation in responses to conventional antidepressants is a recognized limitation of evidence-based pharmacotherapy for MDD. Shariq et al. (2018) reported that cytokine blockade effectively improved the therapeutic efficacy of MDD patients with immune dysfunction, which cannot be achieved by conventional antidepressants alone. Another large-scale, self-controlled study including 18,241 RA-associated depressed patients showed that 20% to 40% of patients who received biologics improved their depressive symptoms after 1 year. Patients with pre-existing depressive symptoms who received biologics for the first time had a lower rate of response to treatment (Matcham et al., 2018). In addition, females, younger ages, and lower baseline HAMD scores were positive factors for improving the response rate to biological disease modifying anti-rheumatic drugs (bDMARDs; Miwa et al., 2018).

It should be noted that bDMARDs are not an absolute advantage in the effect of RA-associated depression, some studies have reached the opposite conclusion. A randomized controlled trial involving 90 patients (Yayikci and Karadag, 2019) indicated that bDMARDs were not better than conventional synthetic disease modifying anti-rheumatic drugs (csDMARDs) for the treatment of RA-associated depression. Compared with methotrexate, leflunomide, and hydroxychloroquine, bDMARDs are associated with high rates of depression, anxiety, and suicide in RA patients (Pinho de Oliveira Ribeiro et al., 2013). These results showed that the efficacy of biological agents for RA-associated depression is controversial, but the specified types of biological agents are not clear in these studies, which may be one of the reasons for the controversial conclusions. Of course, there are also some studies on specific biological agents that may provide more valuable evidence.

### 4.2. Anti-TNF

The levels of TNF in RA patients with depression generally rise (Köhler et al., 2017; Enache et al., 2019). Compared with those who do not respond to anti-TNF treatment, those who do respond to it take a lower risk of depression (Deb et al., 2019). Accordingly, depression may be a predictor of no response or a poor response after 3–6 months of anti-TNF or methotrexate treatment of RA. Infliximab was the first anti-TNF drug used in RA. Early studies found that infliximab could reduce disease activity and improve depression in RA patients (Michelsen et al., 2017). In a 2020 systematic retrospective meta analysis of four randomized controlled studies, infliximab did not have any therapeutic effects on depressive symptoms in RA patients (Bavaresco et al., 2020), and it even seemed to induce suicidal tendencies in a subset of RA patients according to a French retrospective cohort study (Thillard et al., 2020).

### 4.3. IL-6 antibody

IL-6 antibody may have a positive effect on mental health in RA patients. Tocilizumab was the first humanized anti IL-6 receptor monoclonal antibody approved for treating RA refractory to methotrexate or TNF inhibitors. The weekly use of tocilizumab *via* subcutaneous injection has been widely claimed to improve depression in RA patients (Figueiredo-Braga et al., 2018; Tiosano et al., 2020). In contrast to tocilizumab, which targets the IL-6 receptor, sirukumab, and siltuximab directly antagonize IL-6 and block its function. Two-phase double-blind placebo-controlled trials to evaluate the efficacy of sirukumab and siltuximab in RA patients with depression showed that both drugs could improve depressive symptoms, even in patients who did not respond to RA treatment (Sun et al., 2017). However, safety may be a considerably important issue. In the phase 3 double-blind sirukumab study, the respective incidences of adverse events and serious adverse events were 93.4% and 7.4% (Takeuchi et al., 2018).

In summary, compared with non-biological therapies, the efficacy of biological agents in RA patients with depression is still controversial. They may even be associated with more severe depression, anxiety, and suicidal tendencies. Moreover, biotherapies may also lead to adverse effects such as tumors, abnormal blood parameters, infection, and allergy. Hence, the value of biological agents for the treatment of RA-associated depression requires further research.

## 5. Conclusion and prospects

RA can be associated with various comorbidities, among which depression has attracted much attention due to its high incidence and seriousness. The etiology and pathological mechanism of RA- associated depression are complex, in addition to somatic symptoms, drugs, diet, and exercise habits, vitamin D deficiency, Mg deficiency, abnormal testosterone levels, social support, and RA disease activity itself may induce or aggravate the depression, resulting in a vicious circle of “RA-depression-adverse outcomes-social and economic burden”. Immune imbalance and inflammatory stimulation are important pathological mechanisms leading to the bidirectional association between RA and depression. Taking these factors into consideration when choosing a treatment regimen will help with disease remission. At present, some studies have attempted to use biological agents for the efficacy of RA-associated depression, but there is no consensus. Doctors should be alert to the possible risks of biological agents. In the future, larger sample, multi-center, higher-level evidence-based studies related to biologics are needed to provide high-quality evidence for clinical decision-making pertaining to biologics for treating of RA-associated depression.

## Author contributions

NL and WY wrote the manuscript. RS and LZ identified and retrieved the original documents. XW drew the figure. DQ and ZL revised the manuscript. JP raised the idea for the article. All authors contributed to the article and approved the submitted version.
